# Training Behaviors and Periodization Outline of Omnivorous, Vegetarian, and Vegan Recreational Runners (Part A)—Results from the NURMI Study (Step 2)

**DOI:** 10.3390/nu15071796

**Published:** 2023-04-06

**Authors:** Katharina Wirnitzer, Derrick Tanous, Mohamad Motevalli, Christian Raschner, Karl-Heinz Wagner, Gerold Wirnitzer, Claus Leitzmann, Thomas Rosemann, Beat Knechtle

**Affiliations:** 1 Department of Research and Development in Teacher Education, University College of Teacher Education Tyrol, 6010 Innsbruck, Austria; 2 Department of Sport Science, University of Innsbruck, 6020 Innsbruck, Austria; 3 Research Center Medical Humanities, University of Innsbruck, 6020 Innsbruck, Austria; 4 Department of Nutritional Sciences, University of Vienna, 1090 Vienna, Austria; 5 adventureV & change2V, 6135 Stans, Austria; 6 Institute of Nutrition, University of Gießen, 35390 Gießen, Germany; 7 Institute of Primary Care, University of Zurich, 8091 Zurich, Switzerland; 8 Medbase St. Gallen, Am Vadianplatz, 9001 St. Gallen, Switzerland

**Keywords:** plant-based, half marathon, marathon, recreational athlete, training, race, periodization, running

## Abstract

Runners train for long-distance competitions based on underlying motivations, which may be similar to individual dietary motivations (e.g., well-being and performance). Fundamental training differences may arise in recreational runners following different diet types (omnivore, vegetarian, vegan) considering possible motive variations. Following a cross-sectional design, distance runners completed a survey (online), including a thorough assessment of training behaviors with generic training details and periodization specifics in three phases: 1. an intermediary and rebound stage, 2. a main preparatory stage, and 3. a main event stage (tapering or interim event level/s). Kruskal–Wallis and chi-squared tests were used in the statistical analysis. A total of 245 fit recreational runners following omnivore (*n* = 109), vegetarian (*n* = 45), and vegan diets (*n* = 91) were included. Significant differences in the initial running motivation were found across dietary subgroups (*p* = 0.033) as well as for current motivations (*p* = 0.038), with vegetarians being the least health motivated (27% and 9%, respectively). No differences in each of the specific periods were found between diet types across the outline (*p* > 0.05). The present evidence shows that there is a lack of fundamental training differences based on recreational runners following different generic types of diets. The results of the present investigation may be especially relevant for future studies on safety, sustainability, and performance-enhancing dietary practices among athletes.

## 1. Introduction

Running is considered a healthy physical activity for adults, with various benefits for cognitive and physical function, that likely aids in the prevention of lifestyle-induced diseases [1]. People run long distances for various reasons (e.g., fun and tangible rewards) alongside or regardless of the health benefits [2]. In addition, runners follow their current diet type for various purposes, including but not limited to performance, health and well-being, religion, taste, the environment, and animal welfare [3,4]. Thus, recreational runners adhering to general diet type categories may have different motivations for running, which could underlie differences in training behaviors [4].

In previous studies, diet type has been associated with a wide variety of factors such as athletic performance [5], attitudes towards food [6], physical recovery [7], and especially with outcomes of health [8,9]. An individual’s dietary choices are well known to have an influence on many different aspects of an individual’s life, including but not limited to health behavior (e.g., physical activity levels) and social derogation [6,10,11]. The omnivore diet (inclusive of all food types) is by sheer prevalence the leading general type of diet in the world [12]. The Western omnivore diet is often composed of a majority of weekly calories from non-human animal-based sources (typically chicken, pig, cow, or cow by-products, including milk and dairy products) [13,14]. Likewise, with the exemption of meat, the Western vegetarian diet may also predominantly consist of animal-based products (e.g., eggs, yogurt, butter, cheese, and honey) [13,14]. However, any diet may be considered “plant-based” if fruit, vegetable, and grain consumption, for example, exceed 50% of caloric intake [15]. For distance runners switching to a vegan diet (100% plant-based), a noteworthy concern is sufficient caloric consumption and the use of dietary supplements in order to avoid specific micronutrient deficiencies (e.g., vitamin B12, vitamin D, and zinc) [16], which could be challenging considering meal planning alongside training, working, family or social life, and adhering to regular rest and recovery times [15,17]. However, plant-based and especially vegan diets are deemed appropriate for athletes and have been associated with advanced benefits among distance runners [16,18,19].

Training for running distance races with the basis of the periodization technique has been recommended since as early as 1999 [20]. To date, runners typically prepare for a main race with a series of three general training periods of stacking complexity (intermediary, main preparation, and race-focused) and thus follow an overall training duration (typically 3–4 months) [21]. Considerable variation in the FITT (Frequency, Intensity, Time, and Type) principle occurs between recreational runners within training periods, and the amount of focus applied to each training types is related to the individual’s specific training strategy [22,23]. Likewise, recreational runners typically participate in other physical activities parallel to running as the running movement occurs dominantly in the sagittal plane [24,25]. Therefore, frequent long-distance running bouts contribute to chronic postural, flexibility, and strength imbalances (e.g., hip flexor muscle groups), and participation in an array of physical activities may help to balance muscular tension [24,25]. Considering the intricate background of running training (e.g., high-intensity interval training) as well as performing safely in long-distance races, especially for injury avoidance and adequate nutrition, professional support is a critical recommendation for recreational athletes [3,21,24]. In a previous investigation, it was concluded that differences in running and training motivations between recreational runners following different diet types may help to explain possible training behavior differences [5]. Running motivations may, however, change over time or be different for competition in athletes and may even come secondary to motivations for other sports [26]. As training for long-distance races is the foundation of the recreational runner lifestyle [27,28,29], analyzing possible differences between diet types could enhance the explanation for running performance or even potential health risks. Nevertheless, only a few studies have been performed on food choice and/or diet type and training behavior among recreational runners [5,30,31,32].

To date, however, no study has investigated a comprehensive assortment of training behaviors and motives among healthy and fit recreational distance runners following plant-based (vegetarian, vegan) or animal-based diets (omnivore, vegetarian). Therefore, this investigation is the first to assess the motivations behind training and periodization of recreational runners based on following an omnivorous, vegetarian, or vegan diet. Considering the results of a previous NURMI investigation with a more general participant sample [5], this study was performed to test the hypothesis that there are fundamental training differences between recreational runners capable of running at least a half-marathon distance based on following an omnivorous, vegetarian, or vegan diet type.

## 2. Materials and Methods

The Nutrition and Running High Mileage (NURMI) Study protocol [33] was submitted to the ethical board of St. Gallen, Switzerland, and accepted in May 2015 (EKSG 14/145). The NURMI study trial registration was completed retrospectively (ISRCTN73074080). Involving a cross-sectional design, the NURMI study was carried out in three steps. Further details regarding the NURMI study Step 2 methods have been published previously [4,9,34,35,36,37].

The main subjects targeted for enrollment in this study were recreational runners from the countries of Austria, Germany, and Switzerland. Subjects were recruited through various sources, including social media sites, marathon event websites, personal contacts, runner communities online, and email subscriptions to runner magazines of health, lifestyle and nutrition, sports trade fairs, and plant-based lifestyle and diet. In Table 1, the characteristics of the recreational runners are shown.

All subjects who took part in the NURMI study received a written explanation of the study procedure and completed the informed consent before enrollment. Over a period of 11 months, subjects completed an online survey that was available in German and English from www.nurmi-study.com. The survey was primarily based on physical health and food frequency, which included a basic allocation to a focal running area (well-being, hobby, competition) corresponding to the subject’s respective running, exercise, and racing motives, and complimentary sports activities.

Fulfilling the following four inclusion criteria was essential for complete study participation: written informed consent, aged 18 years or older, Step 2 survey completed, and participation and completion of a half-marathon event (or longer distance) within the last two years. Control questions were implemented throughout different sections of the survey in order to verify reliable answers for running activity (including motives, performance history, and training details) as well as diet type; if the datasets submitted were contradictory, lacking critical answers, or conflicting, they were excluded from the investigation.

A total of 91 subjects had not completed an event of at least a half-marathon distance but had completed a 10 km race, and considering the high motivation of these subjects by their accurate and appropriate answers, recreational 10 km runners were included as an additional comparator group due to the high-quality data provided. Moreover, subjects must have chosen a race (long distance preferred) for the “NURMI running event” (i.e., (ultra-)marathon or half-marathon distance), which they trained for and completed in Step 3 (the main NURMI study linked Steps 2 and 3) [33].

The subjects were classified into three dietary subgroups (with the minimum adherence of four weeks): omnivorous (commonly known as mixed or “Western diet”, with no dietary restrictions), vegetarian (no meat/fish, but dairy and egg products) and vegan (no products from animal sources at all, inclusive (processed) meat, fish/seafood/shellfish, dairy products, eggs, honey) [16,38]. Analysis of control questions showed that 29 runners (11.8% of the study sample) had to be shifted to other dietary subgroups: 5 vegan runners (2 to omnivores and 3 to vegetarians) and 24 vegetarian runners (all to omnivores). Despite this, 88.2% of the total sample provided a correct self-report regarding their kind of diet.

In addition, this study included a body mass index (BMI) procedure to exclude subjects with a BMI ≥ 30 kg/m^2^ based on the health recommendation by the World Health Organization (WHO) [39,40]. The BMI procedure was therefore used to control for the subjects having a minimum health status associated with a minimum fitness level, as safely reducing body weight requires other initial health protecting and weight management strategies besides running.

**Figure 1:**
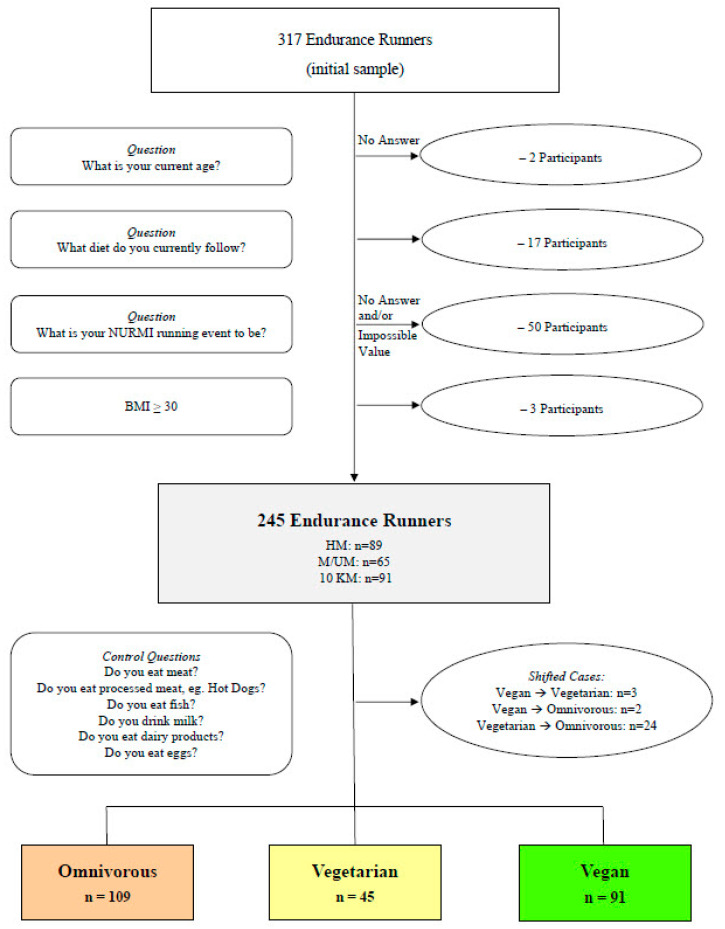
Study enrollment flowchart and dietary subgroup categorization. BMI—body mass index. HM—half-marathon; M/UM—marathon/ultra-marathon; 10 km—10 kilometers.

In Figure 1, the final subjects’ (*n* = 245) categorization into the dietary subgroups is shown along with the flow of study enrollment. Considering that ultra-marathon distances vary, the shortest distance that was completed by any subject in this study was 50 km, and the longest distance completed was 160 km.

The recreational runners’ periodization outline and training details were summarized based on specified variables related to diet type: motivations for running (well-being, hobby, competition) seasonal (winter, spring, summer, autumn) or time of day (morning, afternoon, evening, night) running preferences (indoor and outdoor); overall training duration (from one month to over one year); source of training advice for running (none, qualified professional, other); additional activities parallel to running (summer and winter); periodization outline, including three periods (1–3) and four training types specified for Period 2 (A, B, C, D) as well as weekly number of runs, weekly distance covered (km), weekly duration (hours), daily distance covered (km), and daily duration (hours) for each period and training type.

Statistical analysis was performed using R software (version 4.2.2 UCRT 2022) from the R Foundation for Statistical Computing (Vienna, Austria; www.R-project.org/; accessed on 15 March 2023). Exploration analysis was conducted with descriptive analyses (including medians and interquartile ranges (IQR) as well as mean and standard deviations (SD)). Box plots were used to display the differences in the weekly number of runs and distance covered (km) as well as daily distance covered (km) by the dietary subgroups. The Kruskal–Wallis test and the chi-square test were used to examine the differences between dietary subgroups. The level of statistical significance was set at *p* ≤ 0.05.

## 3. Results

A total of 317 subjects submitted the questionnaire, and 72 (22.7%) were excluded due to not meeting the inclusion criteria. The final sample was made up of 245 recreational endurance runners (42% male) of several self-reported race distances (NURMI-runners: HM, M, or UM: *n* = 154; 10 km runners: *n* = 91) with a median of 7 years (IQR 7) of experience active in running and racing. Regarding the subjects’ academic qualifications (p = 0.029), 34% had completed an upper secondary education (or equivalent; *n* = 83) or held a university degree or higher (*n* = 83; 34%); however, some subjects had provided no answer for academic qualifications (*n* = 25; 10%), and one subject had no degree. The subjects were from various countries (Austria, Germany, or Switzerland: *n* = 234; Belgium, Brazil, Canada, Italy, Luxemburg, Netherlands, Poland, Spain, or United Kingdom: *n* = 11) and were grouped based on their self-reports of following an omnivorous diet (*n* = 109; 45%), a vegetarian diet (*n* = 45; 18%), or a vegan diet (*n* = 91; 37%).

The vegan runners were mostly female (46% vs. 26% male; *p* = 0.004), whereas the omnivores were mostly male (56% vs. 36% female). The omnivores had the highest BMI with a median of 22.6 kg/m^2^ (IQR 3.61), followed by the vegans with 21.3 kg/m^2^ (IQR 3.21), and the vegetarians had the lowest at 20.8 kg/m^2^ (IQR 3.46) (*p* = 0.001). Regarding the subjects’ training focus, no significant difference was found based on the dietary subgroups (*p* = 0.139). A significant difference was found in the marital status of the runners based on diet type (*p* = 0.029), which showed that the omnivores were the least likely to be divorced (3%) or separated (22%), the vegetarians were most often among the single (38%), and the vegans were most common among the divorced or separated (11%). The traits, sociodemographics, and motives of the runners are provided in Table 1 based on the dietary subgroups; further details on these subjects are given in Part B of the sequenced investigation [41].

**Table 1:** Traits, Sociodemographics, and Motives of Runners Displayed by Dietary Subgroups.

		Total	Omnivore	Vegetarian	Vegan	Statistics
		100% (245)	45% (109)	18% (45)	37% (91)	
**Sex**	Female	58% (141)	36% (51)	18% (26)	46% (64)	χ^2^_(2)_ = 11.25 *p* = 0.004
	Male	42% (104)	56% (58)	18% (19)	26% (27)	
**Age** **(years)**		39 (IQR 17)	43 (IQR 18)	39 (IQR 16)	37 (IQR 15)	F_(2, 242)_ = 2.95 *p* = 0.054
**Body Weight** **(kg)**		65 (IQR 14.2)	68 (IQR 16.7)	62 (IQR 11.3)	64 (IQR 10)	F_(2, 242)_ = 6.86 *p* = 0.001
**Height** **(m)**		1.7 (IQR 0.1)	1.7 (IQR 0.1)	1.7 (IQR 0.1)	1.7 (IQR 0.1)	F_(2, 242)_ = 2.45 *p* = 0.088
**BMI** **(kg/m^2^)**		21.7 (IQR 3.5)	22.6 (IQR 3.61)	20.8 (IQR 3.46)	21.3 (IQR 3.21)	F_(2, 242)_ = 7.03 *p* = 0.001
**Academic** **Qualifications**	No degree or certificate	<1% (1)	/	/	1% (1)	χ^2^_(4)_ = 10.78 *p* = 0.029
	Upper secondary education/Technical qualification/GCSE or equivalent	34% (83)	38% (41)	38% (17)	27% (25)	
	A Levels or equivalent	22% (53)	24% (26)	16% (7)	22% (20)	
	University degree/Higher degree	34% (83)	30% (33)	38% (17)	36% (33)	
	No answer	10% (25)	8% (9)	9% (4)	13% (12)	
**Country of** **Residence**	Austria	18% (44)	21% (23)	18% (8)	14% (13)	χ^2^_(6)_ = 9.05 *p* = 0.171
	Germany	72% (177)	70% (76)	76% (34)	74% (67)	
	Switzerland	5% (13)	7% (8)	4% (2)	3% (3)	
	Other	4% (11)	2% (2)	2% (1)	9% (8)	
**Training** **Focus**	Well-being	9% (23)	9% (10)	2% (1)	13% (12)	χ^2^_(4)_ = 6.95 *p* = 0.139
	Hobby	54% (133)	50% (54)	67% (30)	54% (49)	
	Competition	36% (89)	41% (45)	31% (14)	33% (30)	
**Preferred** **Racing** **Distance**	10 km	37% (91)	34% (37)	33% (15)	43% (39)	χ^2^_(4)_ = 3.47 *p* = 0.483
	HM	36% (89)	36% (39)	44% (20)	33% (30)	
	M/UM	27% (65)	30% (33)	22% (10)	24% (22)	
**Initial** **Motivation for Running**	Well-being	44% (108)	49% (53)	27% (12)	47% (43)	χ^2^_(2)_ = 6.82 *p* = 0.033
	Hobby	56% (137)	51% (56)	73% (33)	53% (48)	
**Current** **Motivation for Running**	Well-being	19% (47)	22% (24)	9% (4)	21% (19)	χ^2^_(4)_ = 10.15 *p* = 0.038
	Hobby	46% (113)	38% (41)	64% (29)	47% (43)	
	Competition	35% (85)	40% (44)	27% (12)	32% (29)	

Note. Results are presented as the percentage (%), total numbers, and median (IQR). χ^2^ statistic calculated by Pearson’s chi-square test and F statistic calculated by the Kruskal–Wallis test. 10 km—10 kilometers. HM—half-marathon. M/UM—marathon/ultra-marathon.

### 3.1. Generic Runner Training Activity

For the subjects’ initial motivation for running, a significant difference was found across the dietary subgroups (*p* = 0.033) in which the vegetarians were most likely to report hobby (73%; *n* = 33), whereas the omnivores (49%; *n* = 53), followed by the vegans (47%; *n* = 43), were more likely to report well-being. Regarding the current motivation for running, a significant difference was found based on the dietary subgroups (*p* = 0.038), with the largest proportions of vegetarians (64%; *n* = 29) and vegans (47%; *n* = 43) running for hobby. For running preferences, no differences based on a particular season for outdoor (*p* = 0.351) or indoor (*p* = 0.669) running were identified for the dietary subgroups. No significant difference was found for the dietary subgroups based on the preferred time of day to run outdoors (*p* = 0.354), where most subjects preferred running outdoors in the morning (31%; *n* = 75). A significant difference was found amongst the dietary subgroups for the favorite time of day to run indoors (*p* = 0.048); the omnivores (20%; *n* = 22) were the most likely to run in the evening (indoors) compared to the vegetarians (9%; *n* = 4) and vegans (8%; *n* = 7).

**Figure 2:**
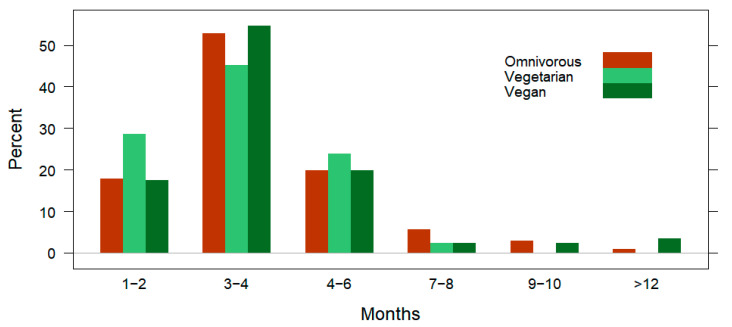
Prevalence of overall training durations (displayed by six clusters) of omnivores, vegetarians, and vegans. Data are presented as the percentage.

**Figure 3:**
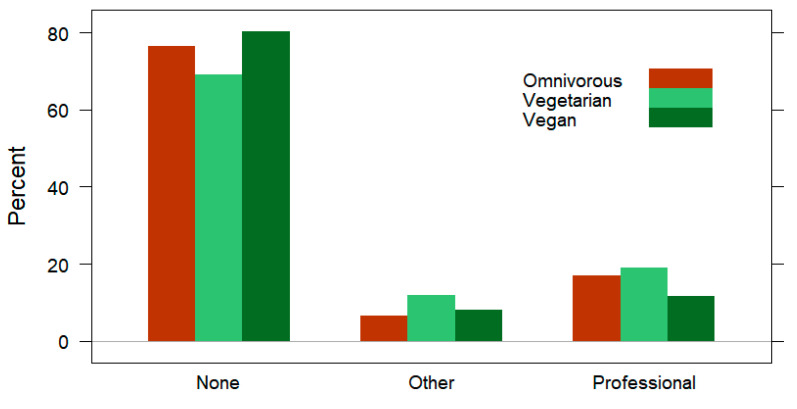
Training advice prevalence (displayed by three clusters) of omnivorous, vegetarian, and vegan runners. Data are presented as the percentage.

In Table 2, the generic training details of subjects are provided by the dietary subgroups. No significant differences were identified between omnivores, vegetarians, and vegans based on the overall training duration spent preparing for the main race (*p* = 0.597; Figure 2) or the source of training advice (*p* = 0.586; Figure 3). The omnivores were the most likely to participate in downhill skiing (24%; *n* = 26; *p* < 0.001) and Nordic skiing (17%; *n* = 18; *p* = 0.026) parallel to running.

### 3.2. Periodization Outline and Recreational Runner Training Specifics

**Figure 4:**
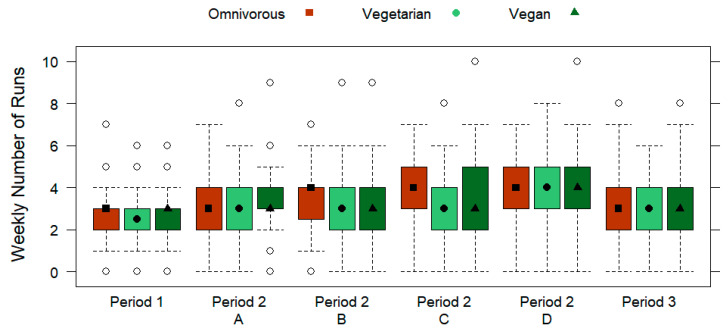
Weekly number of runs (displayed by three training periods, including the A, B, C, and D training types of period 2) of omnivorous, vegetarian, and vegan runners. Data are presented by box plots.

**Figure 5:**
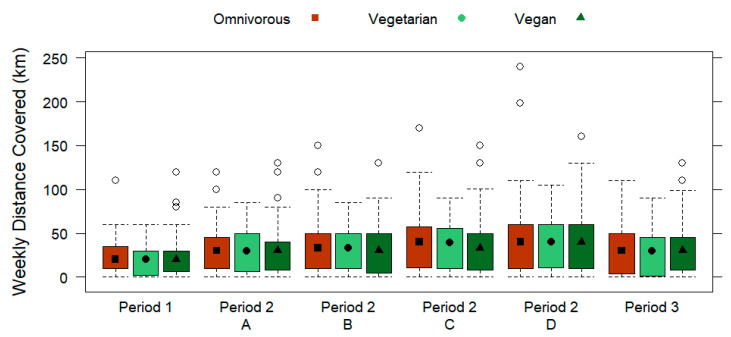
Weekly distance covered (km; displayed by three training periods, including the A, B, C, and D training types of period 2) of omnivorous, vegetarian, and vegan runners. Data are presented by box plots.

**Figure 6:**
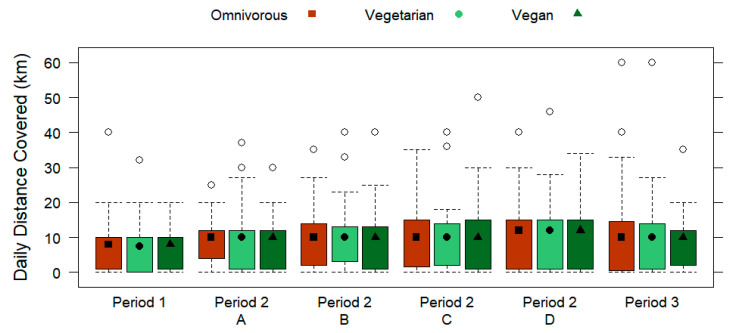
Daily distance covered (km; displayed by three training periods, including the A, B, C, and D training types of period 2) of omnivorous, vegetarian, and vegan runners. Data are presented by box plots.

In Table 3, the periodization outline of the runners is displayed by the dietary subgroups. No significant differences were found for weekly number of runs for any training period based on the dietary subgroups (*p* > 0.05), which is shown in Figure 4. From the total sample, the subjects ran most frequently during the week for training types C (4 runs/week; IQR 2) and D (4 runs/week; IQR 2). Figure 5 and Figure 6 display the weekly running distance covered (*p* > 0.05) and the daily running distance covered (*p* > 0.05) by omnivorous, vegetarian, and vegan runners, respectively, with no significant differences for any training period. Overall, the subjects ran the furthest weekly distance during training type D (39.5 km/week ± 35.8), which was also over the longest duration (5.95 h/week ± 5.39). Furthermore, no significant differences were found for the dietary subgroups regarding the daily running distance covered in kilometers (*p* > 0.05) or the daily running duration in hours (*p* > 0.05) for any training period. Out of the total sample, the most daily running distance covered (10.7 km/day ± 8.31) and duration (0.5 h/day ± 0.39) was for training type D.

## 4. Discussion

The NURMI study Step 2 was designed with the objective of analyzing long-distance runner training behaviors based on various subgroups (sex, preferred race distance, diet type); this investigation was performed to explore the training and periodization differences of omnivorous, vegetarian, and vegan recreational runners capable of running at least a half-marathon distance. The most important findings include (a) the vegan diet was more prevalent in females, and more males followed an omnivorous diet; (b) omnivores had the highest BMI, and vegetarians had the lowest; (c) significant differences were found for the initial and current running motivations based on diet type, where vegetarians were the least motivated for well-being purposes; (d) generic training details were similar based on the dietary subgroups, however, the omnivores were the most likely to participate in downhill and Nordic skiing activities parallel to running. However, the key finding is that no training differences in the periodization outline were identified based on the dietary subgroups.

The present investigation verifies the hypothesis that there are no fundamental differences in training among healthy and fit recreational distance runners based on following an omnivore, vegetarian, or vegan diet type. The lack of key differences in training habits may be due to the constitution of the sample—a recreationally active population—since athletes represent the active lifestyle rather than a devoted dietary lifestyle. Recreational athletes in Europe have been found to have enriched health consciousness compared to the normal population [9,9,33,34,35,42], thus suggesting that the potential effect of diet on training habits is likely to be decreased if not null.

From the history of populations following vegetarian diets [42,43], this investigation found, consistently, that the vegan diet prevalence was more common among females. This finding may be related to the cultural misunderstanding that the consumption of animal flesh is the underlying element of male power, strength, domination, and reproductive potency [43]. However, the relatively few studies comparing strength performance based on omnivore, vegetarian, and vegan diets have not found any consistent relationships [44]. In addition, the world’s largest nutritional organization—the American Academy of Nutrition and Dietetics—has maintained a stable position regarding vegetarian nutrition since 1988 [45]: well-planned vegetarian (notably vegan) diets are healthy and nutritionally sound for any person (male, female, infant/child, adult/older adult, pregnant/lactating, athlete) [16]. Interestingly, however, equal proportions of the sexes following the vegetarian diet (i.e., mainly characterized by the specific exclusion of flesh foods) were found in the present sample. This result may be due to the fact that the male subjects of the present sample were healthy and fit recreational distance runners who appear more likely to follow a vegetarian diet in particular [18]. Furthermore, several subjects in the present investigation had to be shifted from their self-reported dietary subgroup due to misreporting. The vast majority of these misreports (86.2%) were omnivorous participants selecting vegetarian diet, which may be due to the desire of these participants to consider themselves among the plant-based dieters but struggling with initial behavior change [46,47].

Considering that the subjects had a similar height regardless of the diet type they followed, the body weight and BMI differences across the diet type subgroups appears to be particularly related to body composition [39,40]. This investigation found that the omnivores had the highest BMI compared to vegetarians and vegans, which is consistent with the previous large-scale observational studies based on similar dietary subgroups such as the EPIC-Oxford study or the Adventist Health Study 2 [48,49]. However, regarding the nature of the present investigation, which focused on recreational endurance athletes, the present sample was more refined, and subjects were highly physically active, which is critically reflected by the periodization outline (Table 3). Therefore, this may be why the omnivorous subjects of the present sample had a BMI within the normal range [50], as a previous study with a massive sample has shown that omnivores are, on average, within the pre-obese category [39,51]. In addition, considering the current investigation excluded subjects with an obese BMI, this may have benefited the resultant BMI of the omnivorous subgroup (22.6 kg/m^2^) most heavily. Considering that animal products such as milk and cheese are energy dense and mostly composed of protein, fat, cholesterol, and saturated fatty acids [52], the resultant vegetarian BMI (20.8 kg/m^2^) may also have benefitted from the BMI exclusion criteria of the present study, but likely less than the omnivores. While the resultant vegetarian BMI was within the normal range overall, some subjects may have had an underweight BMI, which could be potentially dangerous for health due to malnutrition [17,39]. Considering long-distance running expends a tremendous amount of calories [35,53], hypovitaminosis may occur in underweight distance runners preventing micronutrient absorption and sustaining nutrient deficiency [54]. Although, in running performance motivated subjects, the optimal BMI has been reported between 19–20 kg/m^2^ [55].

These findings, in connection with the resultant vegan BMI of the present investigation (21.3 kg/m^2^), are likely also related to the individual’s proportion of whole plant-based foods consumed, the consumption of animal products, and the fraction of processed food products consumed [15,50,52,56]. As whole plant foods typically contain plentiful loads of complex carbohydrates high in dietary fiber such as starch, digesting these foods provides a continuous, high-quality energy supply contributing to satiety and a lean physique [57,58]. Processed vegan food products, on the other hand, which may be consumed due to convenience, have been associated with increased coronary heart disease risk and type II diabetes [59]. However, even processed vegan food products (e.g., fake meat or mock cheese) are healthier than real animal products and are more environmentally sustainable [60,61]. A previous study published by the NURMI lab on dietary intake identified that vegans consume significantly more whole plant foods such as beans, seeds, fruits, and vegetables [4], which are positively associated with sustainable healthy body weight and BMI [57]. In addition, it has also been found that vegans consume less processed grains or oils but a higher amount of processed plant-based foods such as dairy alternatives, which may be associated with the present sample having a higher BMI than the vegetarians [4]. However, given the high demand of physical exercise in the runner training schedule, regularly consuming energy-dense minimally processed foods such as nut butters (almond, peanut, hazelnut) are recommended in moderation for plant-based athletes to support nutritional needs [62] in addition to adequate consumption of essential vitamins and minerals (especially for females) [63,64].

The current investigation was on recreational runners, who are, based on the aspect of “recreation,” well known to partake in running for hobby or leisure purposes [65]. This link may explain why all subjects, regardless of diet type, mostly reported hobby as their initial motivation for running. Considering that personal dietary motivations likely influence behavior [66] and that personal running motivations likely influence exercise training behavior [2,5], it is interesting that the vegetarians of the present sample were significantly less motivated than the omnivores or vegans regarding running for well-being purposes. In another study on athletes, it was found that vegetarians are highly motivated to follow their current diet based on health and well-being purposes [67]. Therefore, it seems that when it comes to running recreationally, vegetarians are more motivated (initially and currently) to run for hobby or fun than well-being, possibly based on the assumption that their diet (e.g., by the lack of flesh foods: meat and fish/shellfish) is sufficient for their health as a single factor [68]. Vegetarians, however, have still been found to have high rates of hemorrhagic stroke and non-communicable disease [69]. Vegans, on the contrary, have the lowest rates of ischemic heart disease (the world’s leading killer) and most non-communicable diseases [69]. Although, the vegan diet is also limited as a single factor for health [68], which was indicated by a study on bone fractures in which people with a BMI <22.5 kg/m^2^, inactive people, or women following vegan diets had a significantly greater risk of experiencing a bone fracture [70]. However, that investigation included relatively few vegans with no stratification of dietary quality (i.e., proportion of whole vs. processed plant food consumption) and was based on a predominantly white-Caucasian population of mostly middle-aged women (77%) from the UK [70].

The underlying motivation for an individual to follow a specific diet type may be a higher priority for individuals versus the motivation for running [71], especially for people following vegetarian or vegan diets with the principal concern of adherence as ethics and morality [43]. For example, another study found that omnivores were the least likely to follow their diet for the protection of the environment or animals, with a high statistical significance [72]. Although, the present sample on athletes likely includes a higher proportion of people who are especially motivated to choose foods for competition or sporting performance reasons compared to the general population [4,72]. Considering that competition is not experienced by the individual at the start of running (as a physical activity or physical exercise), the competition motivation (and thus being part of a larger sense of community) [73] was unimportant at the start of running regardless of diet type. This link may explain why competition was found to be the predominant current motivation for running of the omnivorous subgroup in this investigation. Although, another investigation on recreational athletes found that omnivores and vegans are significantly more likely than vegetarians to follow their diets for sports performance reasons [4]. The topic of omnivore versus vegan nutrition in sports performance is thus of high interest to many populations, including but not limited to athletes, sport scientists/exercise scientists, exercise physiologists, doctors of sports medicine, fitness trainers, or physical therapists [74]. Part B of the present investigation includes further details regarding the impact of running training and the long-distance race performance comparison based on the subjects following omnivore, vegetarian, and vegan diets [41].

A high prevalence of dietary adherence for health and well-being purposes (≥78%) was previously identified among omnivore, vegetarian, and vegan subgroups [4]. However, concerning the training focus, the initial motivation for running, and the current motivation for running from the present sample, well-being was consistently the least prevalent motive across dietary subgroups. Omnivorous nutrition, on the one hand, is limited for maintaining a sustainably healthy lifestyle in the long-term, as omnivores are highly likely to develop preventable chronic, non-communicable diseases across the lifespan, which result in extensive years of disability leading up to premature mortality [75,76,77,78,79,80]. Accordingly, highly physically fit young men following omnivorous diets have been observed to have severe cases of coronary artery disease [81]. On the other hand, omnivorous nutrition has also been shown to benefit health among omnivores with non-communicable diseases [82]; however, the prerequisite is a very high proportion (at least 70–80%) of whole plant foods making up the daily diet [57].

For the categories of generic training behaviors, only significant differences in the participation of downhill skiing and Nordic skiing were observable across dietary subgroups; the vegans were the least participatory in downhill and Nordic skiing, followed by the vegetarians. While the overall participation in winter sports activities was rather low (≤14% for each activity) among the subjects included in this study, the lack of interest among the participants with vegetarian dietary patterns may be related to climate change affected environments [83,84]. In parallel, the vegetarian, but especially vegan dieters displayed significantly deeper educational backgrounds compared to the omnivores, which is consistent with previous reports [43]. However, a larger sample size may be necessary to explicate the relationship between diet type and participation in winter sports parallel to running. Considering the lack of background data on the topic (differences in recreational runner diet type and participation in skiing), it is not possible to draw links with previous research. Thus, given the exploratory nature of the present study design, it is possible that differences were found across dietary subgroups in participation of these sports activities due to random chance events [85]. Furthermore, considering that the training focus across dietary subgroups was similar, this may explain why no differences in the overall training duration were found as well as the source of training advice or participation in summer sports activities parallel to running. Regarding the overall training duration, the majority of subjects (53%) trained for three to four months before racing and only a small proportion of the subjects trained for more than six months (8%). This finding may be due to the fact that the subjects in this study were not professional runners [21]. Three to four months of overall training duration before a long-distance race has been a viable recommendation for best performance and reducing the likelihood of injury or adverse consequences to running [86]. In connection, following a qualified professional for running training advice was only reported by 15% of the present subjects. In other studies on recreational runners, it has been found that only a minor proportion of these athletes follow professional advice [87,88]. One explanation for this low occurrence of professional guidance may be due to the activity of running being a natural form of movement that develops from childhood [89]. Therefore, runners may naturally feel that they know how to train independently for running half-marathon, marathon, or even ultra-marathon races [74,84,89]. In connection, common occurrences of overuse injuries have been reported among recreational distance runners [90].

Consistently, no significant differences were found for any analysis of the periodization outline based on the dietary subgroups. These results appear to indicate that diet type is mostly unrelated to training behavior among fit distance runners; however, this may be connected to the present subgroups having a similar training focus and the recreation aspect. In another study with a small sample on recreational omnivore, vegetarian, and vegan runners, no differences in training frequency per week or running time per week were found [30]. In Step 1 of the NURMI study, which focused on a gross sample of recreational runners, diet type differences were found in training alone or with a professional and the overall training duration, but the total training volume was similar between omnivores, vegetarians, and vegans [5]. Overall, fundamental differences are negligent in the training behaviors of recreational runners following omnivore, vegetarian, or vegan diets.

Regarding the current results within the specific periods, the initial period (intermediary and rebound stage) is characterized by being transitionary, which is the time duration and basic exercise that occurs at the initiation of running or after completing a main race. From the total sample, the results indicate that the subjects cover a total distance of 22.4 km per week during Period 1 with about three runs per week and up to 28 min per run. This result appears to contrast findings on elite runners; however, this may be due to the heterogeneity amongst recreational runners [21]. The progression of training from Period 1 to each training type of Period 2 shows a successive increase in total running volume through building a training foundation with general running aspects (30.2 km per week with three runs and up to 44 min per run) to specialized race-specific foci (39.5 km per week with four runs and up to 53 min per run), which is consistent with previous reports [91]. As Period 2 is the main preparatory stage, a considerable drop is visible in the training volume over the progression to Period 3. This finding is comparable with previous results, as tapering is a process of reducing the training load before the main competition and has been associated with improved performance [92].

Limitations of the present investigation are predominantly based on the cross-sectional study design; however, the NURMI study is Europe’s largest running study ever performed, including a comprehensive outline of training behaviors among distance runners. Due to the cross-sectional design, the surveying method was the ideal preference to reach a wide variety of subjects, although it was well known that over/under reporting is a common occurrence for such methods. To reduce the effect of misreporting, different survey sections contained critical questions in order to verify consistency within reports. Another limitation is the exploratory characteristic of the NURMI study, which does not allow for deeper explanations regarding the specific differences found across dietary subgroups in Nordic or alpine skiing. However, the NURMI study is unprecedented within the cross-cut disciplines of athletic performance and plant-based nutrition or vegan dietary analyses. A strength of the present investigation is the homogeneity of various characteristics across dietary subgroups, such as age, height, country of residence, training focus, and preferred race distance, allowing for a coherent interpretation of the exploratory analyses.

## 5. Conclusions

This is the first study to analyze a thorough outline of training behaviors among healthy and fit recreational distance runners adhering to omnivore, vegetarian, and vegan diets. Key findings indicate that there are no fundamental differences in the training behaviors between recreational runners based on following an omnivorous, vegetarian, or vegan diet, which is mainly suggested to be a key part of the active lifestyle rather than a general dietary lifestyle. Experts of running, trainers, coaches, and other professionals in sports and nutrition may find the results particularly useful when advising clients who follow plant-based, vegetarian, and vegan diets. Considering the importance of diet type for individuals and especially among recreational distance runners, the results of the present investigation may be especially relevant for future research within the area of cardiovascular safety, long-term health and sustainability, and performance-enhancing dietary practices among athletes.

## Figures and Tables

**Table 2:** Generic Training Details of Subjects by Dietary Subgroups.

		Total	Omnivore	Vegetarian	Vegan	Statistics
		100% (245)	45% (109)	18% (45)	37% (91)	
**Overall Training Duration**						
1–2 months		20% (46)	18% (19)	29% (12)	17% (15)	χ^2^_(10)_ = 8.33 *p* = 0.597
3–4 months		52% (122)	53% (56)	45% (19)	55% (47)	
4–6 months		21% (48)	20% (21)	24% (10)	20% (17)	
7–8 months		4% (9)	6% (6)	2% (1)	2% (2)	
9–10 months		2% (5)	3% (3)	/	2% (2)	
>12 months		2% (4)	< 1% (1)	/	3% (3)	
**Source of Training Advice for Running**						
None		76% (179)	76% (81)	69% (29)	80% (69)	χ^2^_(4)_ = 2.83 *p* = 0.586
Qualified professional		15% (36)	17% (18)	19% (8)	12% (10)	
Other		8% (19)	7% (7)	12% (5)	8% (7)	
**Additional Activities Parallel to Running**						
*Summer*	Biking	53% (130)	57% (62)	51% (23)	50% (45)	χ^2^_(2)_ = 1.21 *p* = 0.546
	Fell/Trail running	31% (75)	32% (35)	31% (14)	29% (26)	χ^2^_(2)_ = 0.29 *p* = 0.867
	Swimming	31% (75)	38% (41)	22% (10)	27% (24)	χ^2^_(2)_ = 4.87 *p* = 0.088
	Rambling	31% (75)	34% (37)	31% (14)	27% (24)	χ^2^_(2)_ = 1.33 *p* = 0.515
	Triathlon	19% (46)	24% (26)	13% (6)	16% (14)	χ^2^_(2)_ = 3.45 *p* = 0.178
*Winter*	Downhill skiing	14% (34)	24% (26)	13% (6)	2% (2)	χ^2^_(2)_ = 19.50 *p* < 0.001
	Nordic skiing	11% (26)	17% (18)	7% (3)	6% (5)	χ^2^_(2)_ = 7.28 *p* = 0.026
	Snowboarding	7% (16)	7% (8)	7% (3)	6% (5)	χ^2^_(2)_ = 0.27 *p* = 0.872
	Backcountry skiing	4% (9)	4% (4)	4% (2)	3% (3)	χ^2^_(2)_ = 0.10 *p* = 0.949

Note. Results are presented as the percentage (%) and total number. χ^2^ statistic calculated by Pearson’s chi-square test.

**Table 3:** Periodization Outline of Runners, Including Specified Training Types Presented by Dietary Subgroups.

		Total 100% (245)	Omnivore 45% (109)	Vegetarian 18% (45)	Vegan 37% (91)	Statistics
**Period 1—Intermediary and Rebound Stage**						
Weekly:	Number of runs	3 (IQR 1)	3 (IQR 1)	2 (IQR 1)	3 (IQR 1)	F_(2, 219)_ = 0.10; *p* = 0.909
	Distance covered (km)	22.4 ± 18.9	23.5 ± 18.2	20.6 ± 17.4	21.8 ± 20.6	F_(2, 219)_ = 0.74; *p* = 0.479
	Duration (hours)	1.21 ± 1.03	1.28 ± 0.99	1.12 ± 0.95	1.18 ± 1.12	F_(2, 219)_ = 0.72; *p* = 0.489
Daily:	Distance covered (km)	7.06 ± 5.86	7.37 ± 6.12	6.86 ± 6.51	6.79 ± 5.21	F_(2, 219)_ = 0.37; *p* = 0.691
	Duration (hours)	0.26 ± 0.21	0.27 ± 0.22	0.25 ± 0.23	0.25 ± 0.18	F_(2, 219)_ = 0.33; *p* = 0.717
**Period 2—Main Preparatory Stage**						
***Training Type A (fundamental basics, generally at low intensity)***						
Weekly:	Number of runs	3 (IQR 2)	3 (IQR 2)	3 (IQR 2)	3 (IQR 1)	F_(2, 219)_ = 0.32; *p* = 0.724
	Distance covered (km)	30.2 ± 24.6	31.5 ± 24	29.7 ± 23.5	28.8 ± 25.9	F_(2, 219)_ = 0.46; *p* = 0.633
	Duration (hours)	4.63 ± 3.76	4.83 ± 3.67	4.56 ± 3.59	4.41 ± 3.98	F_(2, 219)_ = 0.58; *p* = 0.561
Daily:	Distance covered (km)	8.74 ± 6.61	8.8 ± 6.01	9.88 ± 8.65	8.09 ± 6.07	F_(2, 219)_ = 0.58; *p* = 0.561
	Duration (hours)	0.4 ± 0.3	0.4 ± 0.27	0.45 ± 0.39	0.37 ± 0.27	F_(2, 219)_ = 0.55; *p* = 0.577
***Training Type B (progressive training with specialized focus, low and moderate intensities)***						
Weekly:	Number of runs	3 (IQR 2)	4 (IQR 2)	3 (IQR 2)	3 (IQR 2)	F_(2, 219)_ = 0.21; *p* = 0.810
	Distance covered (km)	33.5 ± 27.5	34.9 ± 28.1	33 ± 24.4	32.1 ± 28.5	F_(2, 219)_ = 0.34; *p* = 0.711
	Duration (hours)	4.82 ± 3.96	5.02 ± 4.05	4.76 ± 3.5	4.61 ± 4.1	F_(2, 219)_ = 0.38; *p* = 0.684
Daily:	Distance covered (km)	9.42 ± 7.29	9.4 ± 6.74	10.1 ± 8.46	9.07 ± 7.36	F_(2, 219)_ = 0.22; *p* = 0.802
	Duration (hours)	0.41 ± 0.32	0.41 ± 0.29	0.44 ± 0.36	0.39 ± 0.32	F_(2, 219)_ = 0.25; *p* = 0.777
***Training Type C (pacing strategy, specifications for main event, interval training, moderate and high intensities)***						
Weekly:	Number of runs	4 (IQR 2)	4 (IQR 2)	3 (IQR 2)	3 (IQR 3)	F_(2, 219)_ = 0.48; *p* = 0.616
	Distance covered (km)	37.1 ± 31.1	39.2 ± 31.8	36.2 ± 28.1	35.1 ± 32	F_(2, 219)_ = 0.62; *p* = 0.540
	Duration (hours)	5.65 ± 4.73	5.96 ± 4.84	5.52 ± 4.26	5.33 ± 4.86	F_(2, 219)_ = 0.62; *p* = 0.540
Daily:	Distance covered (km)	9.98 ± 7.86	10 ± 7.37	10.3 ± 8.5	9.8 ± 8.18	F_(2, 219)_ = 0.05; *p* = 0.950
	Duration (hours)	0.41 ± 0.32	0.41 ± 0.3	0.43 ± 0.35	0.41 ± 0.34	F_(2, 219)_ = 0.05; *p* = 0.947
***Training Type D (event trials, main event specialized, moderate and high intensities)***						
Weekly:	Number of runs	4 (IQR 2)	4 (IQR 2)	4 (IQR 2)	4 (IQR 2)	F_(2, 219)_ = 0.04; *p* = 0.965
	Distance covered (km)	39.5 ± 35.8	42.2 ± 40.2	37.4 ± 30.1	37.4 ± 32.8	F_(2, 219)_ = 0.12; *p* = 0.891
	Duration (hours)	5.95 ± 5.39	6.34 ± 6.06	5.63 ± 4.53	5.63 ± 4.94	F_(2, 219)_ = 0.12; *p* = 0.889
Daily:	Distance covered (km)	10.7 ± 8.31	10.7 ± 8.28	11.2 ± 8.92	10.5 ± 8.1	F_(2, 219)_ = 0.08; *p* = 0.922
	Duration (hours)	0.5 ± 0.39	0.5 ± 0.39	0.53 ± 0.42	0.49 ± 0.38	F_(2, 219)_ = 0.09; *p* = 0.918
**Period 3—Main Event Stage (tapering or interim event level/s)**						
Weekly:	Number of runs	3 (IQR 2)	3 (IQR 2)	3 (IQR 2)	3 (IQR 2)	F_(2, 219)_ = 0.16; *p* = 0.848
	Distance covered (km)	32.2 ± 27.7	34.3 ± 29.1	29.7 ± 25.8	31 ± 27.2	F_(2, 219)_ = 0.37; *p* = 0.693
	Duration (hours)	4.41 ± 3.8	4.69 ± 3.98	4.06 ± 3.52	4.25 ± 3.72	F_(2, 219)_ = 0.31; *p* = 0.731
Daily:	Distance covered (km)	9.35 ± 8.7	9.67 ± 9.45	10.1 ± 10.5	8.57 ± 6.51	F_(2, 219)_ = 0.19; *p* = 0.831
	Duration (hours)	0.41 ± 0.37	0.42 ± 0.41	0.44 ± 0.45	0.37 ± 0.28	F_(2, 219)_ = 0.13; *p* = 0.882

Note. Results are presented as the median (IQR) and mean ± SD. F statistic calculated by the Kruskal–Wallis test. km—kilometer.

## Data Availability

The datasets generated during and/or analyzed during the current study are not publicly available but may be made available upon reasonable request. Subjects will receive a brief summary of the results of the NURMI study if desired.
